# Effects of Egg White on the Texture, Physicochemical Properties and Sensory Characteristics of Double Protein Yogurt During Storage

**DOI:** 10.3390/gels11110865

**Published:** 2025-10-28

**Authors:** Yuhang Gao, Rongcheng Li, Jie Pan, Yihan Zhang, Renfeng Gao, Ning Xia, Huajing Liu, Lifeng Wang

**Affiliations:** 1 College of Food Science, Northeast Agricultural University, Harbin 150030, China; 13012010137@163.com (Y.G.); lirungcheng@163.com (R.L.); a10220338@neau.edu.cn (Y.Z.);; 2 College of Resource and Environment, Northeast Agricultural University, Harbin 150030, China; panjie202506@163.com

**Keywords:** egg white, yogurt, Storage, characteristics, flavor

## Abstract

With the growing demand for functional dairy products, integrating dual-animal proteins presents a promising strategy to enhance both nutritional value and functional properties. This study aimed to elucidate the impact of egg white supplementation on the stability, physicochemical attributes, sensory quality, and shelf-life of yogurt. Yogurt samples were prepared by fermenting milk supplemented with 0%, 5%, 10%, 15%, 20%, and 25% egg white, and subsequently evaluated for physicochemical parameters, microstructure, rheological behavior, water-holding capacity, and sensory profiles using an electronic nose and electronic tongue. Results showed that 5% egg white significantly improved yogurt stability after one day of refrigeration, whereas 10% supplementation yielded texture comparable to the control but with enhanced protein content, water retention, gel strength, and microstructural uniformity. Over 14 days of cold storage, a gradual decline occurred in physicochemical and structural parameters across all samples; however, flavor profiles remained largely stable, with no adverse effects on sensory quality except for a mild increase in acidity. These findings highlight egg white as a functional ingredient capable of improving yogurt stability and textural quality without compromising flavor, offering new opportunities for the development of high-protein, dual-animal protein fermented dairy products in the functional food industry.

## 1. Introduction

Yogurt has become an essential component of modern healthy diets due to its distinctive flavor profile and rich nutritional content. It contains abundant food compounds with high nutritional content, including peptides, proteins, calcium, phosphorus, potassium, vitamin D, vitamin B_12_ and riboflavin [1]. Yogurt is commonly produced through the fermentation of milk by lactic acid bacteria under controlled temperature and environmental conditions [2]. The lactic acid bacteria metabolize lactose to produce lactic acid and the resulting pH decrease induces milk protein denaturation, which confers yogurt’s characteristic texture and sour taste [3]. The presence of viable lactic acid bacteria in yogurt supports and modulates gut microbiota balance and also modulates the intestinal immune microenvironment, thereby enhancing host immunity [4].

Egg white is recognized as a high-quality protein source, and is widely utilized in the food industry due to its distinctive functional properties [5]. As the main component of eggs, it constitutes approximately 58% of the total egg mass [6]. Egg white contains more than 40 distinct proteins, with major constituents including ovalbumin (54%), ovotransferrin (12–13%), ovomucoid (11%), globulins (8%), lysozyme (3.4–3.5%) and ovomucin (1.5–3.5%) [7]. Notably, egg white provides a complete profile of essential amino acids that closely aligns with human dietary requirements, thereby exhibiting high digestibility and bioavailability [8]. Furthermore, these proteins demonstrate significant bioactive properties, including antioxidant, antimicrobial, antiviral, and immunomodulatory activities [9].

Multi-protein yogurt is produced through the co-fermentation of two or more protein sources scientifically formulated to achieve complementary nutritional benefits. This type of yogurt offers advantages such as enhanced nutritional value and a richer taste profile. However, the incorporation of additional proteins may also introduce challenges, including undesirable flavors and reduced product stability. Current research on multi-protein yogurt has predominantly focused on plant-based protein, with studies on soy-based yogurt revealing certain limitations in product quality and flavor profiles [10]. Previous studies have found that soy protein is often associated with undesirable off-flavors and may negatively affect product quality [11]. Mousavi et al. [12] found that flaxseed supplementation during 28-day refrigerated storage yielded functional yogurt with satisfactory textural properties. However, limited studies have systematically investigated the effects of egg white addition levels on yogurt quality. In this study, we prepared yogurt as co-fermentation of milk with varying egg white concentrations to comprehensively evaluate the effect on stability and flavor characteristics during cold storage (4 °C). The quality characteristics were systematically analyzed at two storage periods (1 day and 14 days post-fermentation) to investigate egg white-induced modifications in yogurt quality and structural properties. This study provides fundamental theoretical insights for egg white yogurt development while proposing novel perspectives for dual-animal-protein yogurt product innovation.

## 2. Results and Discussion

### 2.1. Color Characteristics of Yogurt

Color is an important sensory attribute influencing consumer perception and acceptance of yogurt products. The color of yogurt stored for 1 day is shown in Table 1, L* value increased from 89.43 (EW_0_) to 97.27 (EW_25_), b* value increased from 0.95 to 4.13. a* values were negative and gradually increasing from −0.60 to −0.28. These results indicate that egg white addition progressively brightened the yogurt color and reduced the green and blue components. The calculated whiteness index (WI), based on L*, b*, and a* values exhibited a continuous increased trend indicating egg white enhanced the brightness and whiteness of the yogurt. Yogurt color is influenced by material color and product structure; the higher color values of egg white yogurt compared to the control may be attributed to may be attributed to egg white–induced alterations in the gel network, resulting in a whiter appearance [13].

As shown in Table 2 the L*, a* and WI values increased after 14 days of refrigeration, while the b* value decreased. This suggests that prolonged cold storage, along with continued fermentation by lactic acid bacteria and the activity of other microorganisms, further influenced the color characteristics of the yogurt [14]. This phenomenon may be related to the relative stability of the protein network formed through interactions between egg white proteins and casein, and influences light scattering properties [15]. As a result, the L*, a*, and b* values of egg white yogurt became similar to those of the control after 14 days of storage (*p* < 0.05). Zhao et al. [16] found in their study on the structural characteristics and gel properties of pea protein isolate–egg white protein mixtures that egg white protein increased the whiteness of composite gels in a dose-dependent manner, primarily due to the inherent whiteness of egg white protein. The addition of egg white to yogurt thus influenced light scattering during storage and affected the color properties.

### 2.2. pH of Yogurt

**Figure 1:**
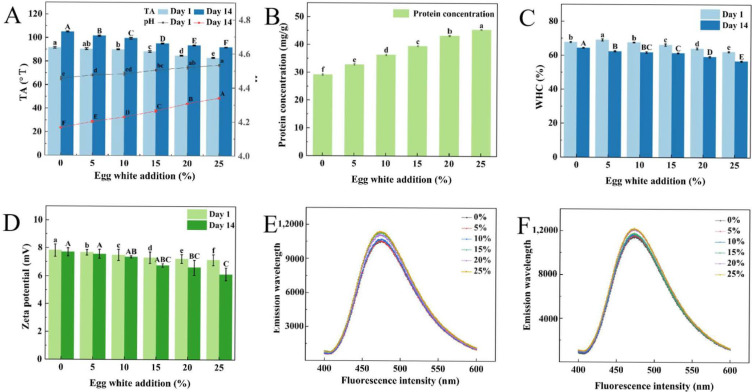
Effect of egg white addition levels on the physico-chemical properties of yogurts during 1-day and 14-day refrigerated storage: pH and TA (**A**), Protein content (**B**), Water-holding capacity (WHC) (**C**), Zeta potential (**D**), Surface hydrophobicity ((**E**): 1-Day, (**F**): 14-Day). Different letters represent significant differences at *p* < 0.05 (*n* = 3).

The formation of yogurt is associated with the pH of the system. Lactic acid bacteria produce lactic acid during fermentation, and the accumulation of organic acids lowers the pH. When the pH drops below the isoelectric point of casein (pH 4.6), the proteins denature and coagulate, forming yogurt. As shown in Figure 1A, increasing egg white content led to yogurt pH increasing, all egg white yogurts exhibiting significantly higher pH values than the control. The control yogurt showed the lowest pH value of 4.45, EW_25_ showed the highest pH of 4.53 (*p* < 0.05); however, all pH values remained below 4.6. The pH trend remained 1 day and 14 days, showing increase with egg white concentration. However, the pH of all samples decreased compared to day 1. The control sample showed the most substantial decrease in pH, whereas higher egg white addition led to a slight pH decrease. Previous studies have reported egg white may suppress the growth of Streptococcus thermophilus, which may be attributed to its relatively higher pH and the presence of antimicrobial components such as lysozyme and avidin [17,18]. Additionally, egg white is rich in protein but lacks lactose, which may contribute to the higher pH observed in the experimental groups compared to the control [19].

### 2.3. Titratable Acidity (TA) of Yogurt

Acidity (TA) is a key parameter for evaluating yogurt quality. During storage, acid formation occurs for microbial metabolism and the degradation of macromolecules [20]. As shown in Figure 1A, TA was decreased with increasing egg white content, with the control yogurt exhibiting higher TA than egg white-supplemented yogurts. This reduction may be attributed to changes in the fermentation environment induced by egg white addition, which influenced bacterial growth and inhibitory acid production, consequently affecting overall yogurt acidity. After 14 days of refrigeration, TA increased in all samples compared to day 1, showing an inverse trend opposite to pH. Specifically, TA values for EW_0_ and EW_25_ increased from 91.16 °T and 83.3 °T (day 1) to 105.1 °T and 92.2 °T (day 14), respectively (*p* < 0.05). Previous studies suggest that although lactic acid bacteria (LAB) continue producing lactic acid during cold storage, certain components may inhibit LAB-mediated acidification and thereby regulate yogurt acidity [21]. These findings demonstrate a consistent inverse relationship between pH and TA in yogurt. Egg white incorporation elevated pH while reducing TA, indicating its influence on the fermentation dynamics.

### 2.4. Protein Content of Yogurt

As shown in Figure 1B, the protein concentration increased progressively from 29.17 mg/g (EW_0_) to 45.45 mg/g (EW_25_) (*p* < 0.05), due to the inherently high protein content of egg white. During yogurt fermentation microbial activity generates various proteolytic enzymes that hydrolyze proteins, leading to partial protein degradation [22]. Previous studies have shown that lactic acid fermentation reduces the relative proportion of ovalbumin and induces mild hydrolysis of egg white proteins [23]. Incorporation of egg white as a primary ingredient also contributed to effective fat control in the final product. Consequently, the resulting egg white yogurt served as a high-protein dairy product and exhibits reduced overall fat content. These combined characteristics make it an ideal choice for health-conscious consumers [24].

### 2.5. Water-Holding Capacity (WHC) of Yogurt

Water-holding capacity (WHC) is a critical parameter reflecting the microstructure and stability of yogurt, directly influencing appearance and texture. As shown in Figure 1C, the WHC of yogurts stored for 1 day initially increased and then decreased. EW_5_ exhibited the highest WHC (69.03%) (*p* < 0.05), indicating enhanced water retention, reduced syneresis, and a more compact gel network that effectively immobilized water molecules [25,26]. The WHC of EW_10_ (67.64%) and EW_0_ (67.68%) were nearly identical, the gel network structures may have similar structural characteristics. However, further increases in egg white content gradually reduced WHC, excessive egg white disrupts the three-dimensional protein network, destabilizing the structure and promoting water loss [27]. After 14 days of storage, all yogurts showed a reduction in WHC compared to day 1, EW_0_ and EW_5_ declining to 58.28% and 56.39%, respectively. This trend indicates a progressive loosening of the gel network and weakened water-binding capacity during storage. Previous research reported that low WHC or whey separation correlates with unstable gel networks and inadequate structural reorganization [28]. The decrease in gel strength and water-holding capacity after egg white addition may be attributed to intermolecular interactions between casein and ovalbumin. Electrostatic attraction at pH values close to the isoelectric point of ovalbumin facilitates the formation of a more compact protein matrix. Additionally, hydrophobic interactions and hydrogen bonding between denatured ovalbumin and casein micelles may promote network cross-linking during fermentation [29].

### 2.6. Zeta Potential Analysis of Yogurt

The Zeta potential (positive or negative) reflects the intensity of electrostatic interactions among protein molecules, with higher absolute values indicating greater colloidal stability [30]. Figure 1D showed the Zeta potential values of yogurt gradually decreased with increasing egg white content, due to lactic acid production during fermentation and decreased the system pH [31]. After 14 days of refrigeration, the Zeta potential values of all yogurts showed a decreasing trend. The reduction was significant with egg white content, due to the metabolic activities of lactic acid bacteria and degradation of macromolecules during storage, which partially neutralized the positively charged proteins. The measured Zeta potential values showed a slight decrease over the 14-day storage period: EW_0_ declined from 7.81 mV (day 1) to 7.70 mV (day 14), EW_5_ from 7.67 mV to 7.56 mV, and EW_10_ from 7.49 mV to 7.35 mV (*p* < 0.05). The positive Zeta potential values indicate net positive charges on protein micelles, could occur when milk fermentation by lactic acid bacteria acidifies the environment, thereby increasing proton concentration and conferring positive surface charges on caseins [11]. This increased proton concentration lowers the system pH, subsequently modifying the net charge of proteins [32]. The reduction in net negative charges weakens interprotein interactions and consequently affects gel network formation [33].

### 2.7. Surface Hydrophobicity (Ho) of Yogurt

As shown in Figure 1E, the surface hydrophobicity of yogurts increased with higher egg white content (1 day), likely resulting from protein unfolding during fermentation that exposed hydrophobic groups [34]. Figure 1F shows the result of 14 days refrigeration, surface hydrophobicity exhibited an increasing trend with egg white addition and showed slightly higher values similarly with day 1 samples. This observation may be attributed to structural loosening of yogurt proteins during extended storage, resulting in increased exposure of hydrophobic residues. Previous studies have demonstrated that a decrease in α-helix content accompanied by an increase in random coil structures contributes to increased molecular disorder and relaxation of protein secondary structures [35]. Such structural unfolding facilitates exposure of buried hydrophobic residues on protein surfaces, consequently increasing surface hydrophobicity [36]. However, excessive exposure of hydrophobic groups has been reported to compromise protein stability, which explains the observed decreases in WHC and occurrence of whey separation in yogurt systems.

### 2.8. Texture Profile Analysis of Yogurt

**Figure 2:**
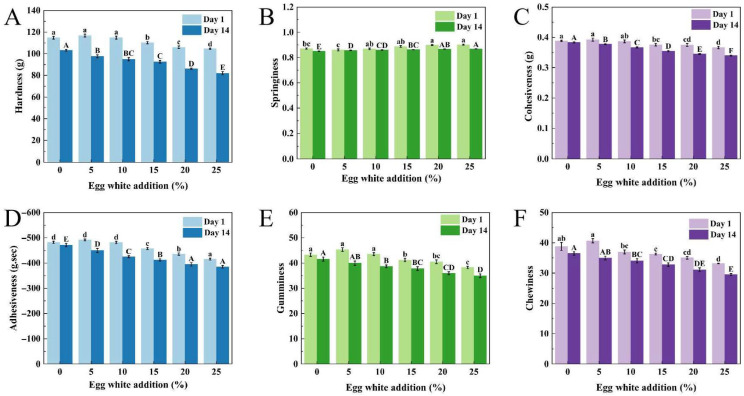
Effect of egg white addition levels on the textural properties of yogurts during 1-day and 14-day refrigerated storage: Hardness (**A**), Springiness (**B**), Cohesiveness (**C**), Gumminess (**D**), Adhesiveness (**E**), Chewiness (**F**). Different letters represent significant differences at *p* < 0.05 (*n* = 3).

As shown in Figure 2, the texture parameters (hardness, cohesiveness, adhesiveness, gumminess, and chewiness) of yogurts stored for 1 day initially increased and then decreased with increasing egg white content. At EW_5,_ all texture parameters reached their maximum values: 116.81 g, 0.393, −491.63 g·s, 45.3, and 40.672, respectively, which were slightly higher than those of the control (EW_0_: 114.78, 0.389, −481.73 g·s, 43.26, and 38.79; *p* < 0.05). This might be attributed to interactions between egg white proteins and casein during fermentation, which promoted the formation of hydrogen bonds, hydrophobic interactions and ionic bridges, resulting in a more compact and stable composite protein network that enhanced textural properties [37]. However, in the sample of EW_10_, all texture parameters were decreased. Springiness exhibited an initial decrease followed by a slight increase, though the differences were not statistically significant. During refrigeration, yogurt acidity significantly increased (Figure 1), leading to weakened protein interactions and subsequent texture deterioration [38]. After 14 days of storage, all textural parameters showed a declining trend. Similar findings have been reported in yogurt studies incorporating zeaxanthin nanoparticles and grape extract [39]. The rearrangement of the gel matrix throughout storage may explain the observed texture degradation [40].

### 2.9. Rheological Properties of Yogurt

**Figure 3:**
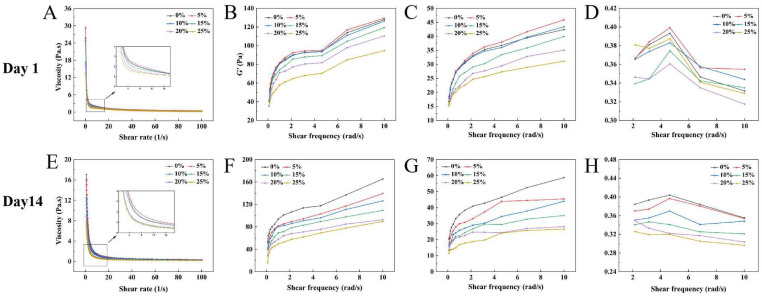
Effect of egg white addition levels on the rheological properties of yogurts during 1-day and 14-day refrigerated storage: Apparent viscosity (**A**,**E**); Storage modulus (G′) (**B**,**F**); Loss modulus (G″) (**C**,**G**); Frequency dependence of tan δ at different time points (**D**,**H**) (*n* = 3).

Viscosity is an important rheological indicator that reflects the internal structural characteristics and dynamic changes of yogurt. All yogurt samples exhibited decreasing apparent viscosity with increasing shear rate (Figure 3A,E), demonstrating the typical shear-thinning behavior of pseudoplastic fluids [41,42]. A significant viscosity reduction was observed between 0.1 s^−1^ and 20 s^−1^, characteristic of non-Newtonian fluids, followed by stabilization above 20 s^−1^ as molecular/particle alignment overcame Brownian motion effects [43]. Figure 3A reveals that apparent viscosity initially increased then decreased from EW_0_ to EW_25_. The initial apparent viscosities were 25.71 Pa·s (EW_0_) and 24.87 Pa·s (EW_10_), with EW_5_ showing the highest value (29.37 Pa·s) (*p* < 0.05). The viscosity of EW_5_ increase may be due to protein–protein interactions and improved water-binding capacity during fermentation [44]. After 14-day refrigeration, apparent viscosity decreased overall, EW_0_ and EW_5_ showing initial values of 17.07 Pa·s and 15.84 Pa·s, respectively: lower than their 1-day. This reduction likely reflects structural loosening of the gel network and weakened water-binding capacity during cold storage.

Within the measured frequency range, all samples in Figure 3 exhibited storage modulus (G′) values consistently higher than loss modulus (G″) values, indicating that the egg white yogurts exhibited dominant solid-like behavior, consistent with typical dairy-based yogurt characteristics [45]. G′ and G″ increased with rising frequency, indicating that the protein interactions within the yogurt matrix were frequency dependent. And the rise in G′ values was attributed to altered intermolecular interactions [46]. From EW_0_ to EW_25_, G′ and G″ initially increased then decreased (Figure 3B,C), with EW_5_ demonstrating peak values. The 5% egg white addition enhanced protein aggregation and reduced system fluidity. Egg white addition led to decreased moduli. G′ and G″ values exhibited decreasing trends from EW_0_ to EW_25_ (Figure 3F,G). The moduli at higher frequencies were greater after 14-day refrigeration compared to day 1 samples, indicating structural weakening of the yogurt gel network during prolonged storage. The tanδ profiles of yogurt samples are presented in Figure 3D,H. Higher tanδ values indicate more viscous/fluid-like behavior, while lower values reflect elastic/solid-like characteristics [47]. All tested yogurt formulations exhibited tanδ values below unity, confirming their predominantly solid-like viscoelastic nature. The results demonstrate that moderate egg white incorporation can effectively enhance the rheological properties of yogurt. However, prolonged refrigeration was observed to increase system fluidity while attenuating semi-solid characteristics.

### 2.10. Secondary Structure Analysis of Yogurt

**Figure 4:**
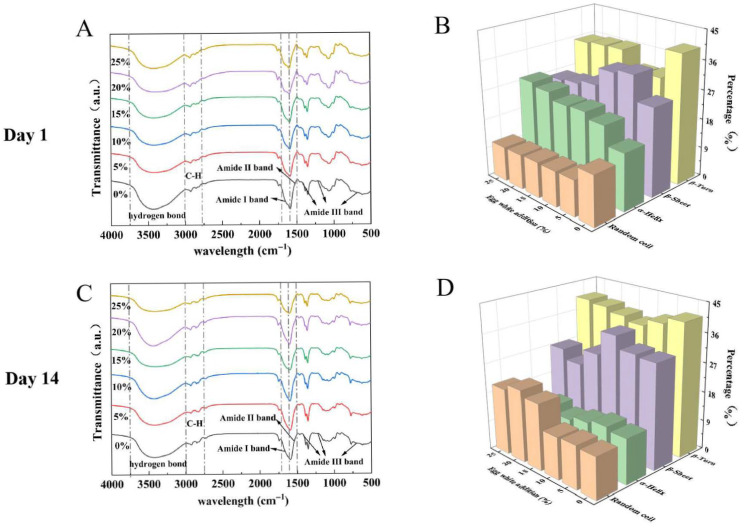
Effects of egg white addition levels on the FTIR spectra (**A**,**C**), secondary structure content (**B**,**D**) of yogurts during 1-day and 14-day refrigerated storage. The *x*-axis represents the name of the secondary structure, the *y*-axis represents the addition amount of egg white, and the *z*-axis represents the content of the secondary structure.

Figure 4A,C demonstrates alterations in absorption peaks in yogurts prepared with varying egg white concentrations. There was a significant decrease in hydrogen bond-related peaks observed in 14-day yogurts compared to their 1-day counterparts, consistent with the water-holding capacity measurements. These findings demonstrate that egg white incorporation significantly influences chemical bond formation in yogurt, with hydrogen bonds exhibiting significant alterations during cold storage. Figure 4B,D shows that in 1-day samples, α-helix content increased from 17.53% to 28.04%, while β-sheet content initially rose and then declined. Random coil structures decreased from 15.35% to 10.70%; β-turn percentages remained lower than controls throughout. These transformations reflect the reorganization of disordered secondary structures into more ordered configurations during protein gelation [48]. The predominance of β-sheets and α-helices in egg white yogurt showed the added egg white proteins did not disrupt the native stable structure [49], but rather interacted synergistically with casein to maintain an ordered secondary structure configuration. At EW_15_, partial conversion of β-sheets and α-helices into disordered β-turns and random coils was observed, likely due to protein overcrowding. However, the majority retained ordered conformations, minimizing structural disruption. After 14 days of refrigeration, significant alterations in yogurt’s secondary structure were observed. The content and trends of β-sheets and β-turns remained largely consistent with day-1 samples, the α-helix content decreased substantially. Concurrently, disordered random coil structures increased from 12.85% to 22.38%; structural modifications indicate that continued fermentation by lactic acid bacteria during cold storage progressively destabilized the protein matrix, ultimately compromising the yogurt’s structural integrity and functional properties [20]. These findings demonstrate that egg white addition promotes more ordered structural organization, and extended refrigeration drives gradual disordering of the secondary structure.

### 2.11. Microstructural Characterization of Yogurt

**Figure 5:**
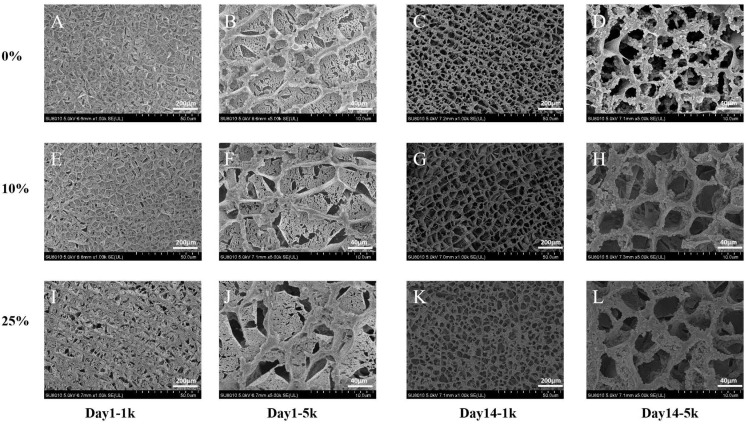
Effect of egg white addition levels on the microstructure of yogurts during 1-day and 14-day refrigerated storage: 0% (**A**–**D**). 10% (**E**–**H**) and 25% (**I**–**L**) (1 k indicates 1000× magnification, 5 k indicates 5000× magnification.) (*n* = 3).

As shown in Figure 5, the microstructures of yogurts refrigerated for 1 and 14 days were comparatively analyzed. EW_10_ (E, F) exhibited a gel network structure similar to EW_0_ (A, B), characterized by smaller voids and a more compact, uniformly distributed porous network. In contrast, EW_25_ (I, J) exhibited significantly reduced structural homogeneity, with a loose gel network with numerous large voids. The compactness of the gel network correlates with protein–protein interactions [50]. After 14-day refrigeration, all samples showed varying degrees of structural loosening. EW_0_ (C, D) maintained a relatively dense and uniform microstructure, while EW_10_ (G, H) developed a homogeneous network with slightly enlarged pores. EW_25_ (K, L) exhibited the most pronounced microstructural degradation with irregular void distribution. Compared with EW_25_, EW_10_ developed a more uniform and compact three-dimensional network. This structural configuration contributes to network stability, explaining the improved rheological properties and higher water-holding capacity [51] observed in our previous analyses.

### 2.12. Aroma and Taste Profiles

**Figure 6:**
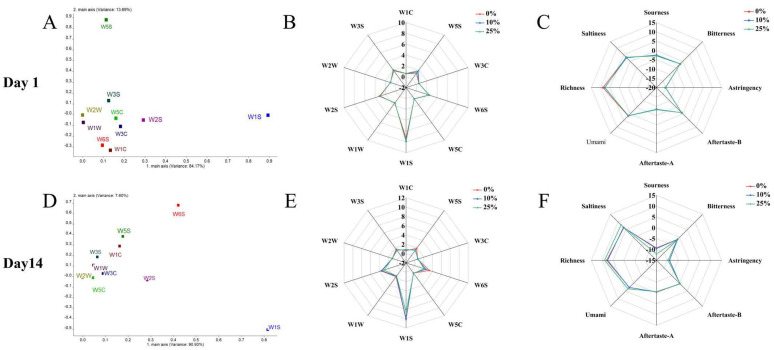
Effect of egg white addition levels on on the electronic nose (e-nose) and electronic tongue (e-tongue) profiles of yogurts during 1-day and 14-day refrigerated storage: Loadings plots of volatile aroma compounds (**A**,**D**), Radar Chart of Electronic Nose Sensor Responses (**B**,**E**), Radar charts of e-tongue sensor responses (**C**,**F**) (*n* = 3).

As shown in Figure 6A, principal component 1 (PC1) accounted for 84.17% of the total variance, while principal component 2 (PC2) explained an additional 13.69%. Together, PC1 and PC2 explained 97.86% of the total variance (>80%), indicating that the first two principal components adequately captured the aroma profile of yogurts stored for 1 day. Sensors W1S (alkanes), W5S (nitrogen oxides), and W2S (alcohols and partial aromatic compounds) exhibited high response values across all yogurt samples. In Figure 6D, the first two principal components (PCs) accounted for 98.53% of the total data variance (PC1: 90.93%, PC2: 7.60%), effectively characterizing the aroma profiles after 14-day refrigeration. Sensors W1S (alkanes), W2S (alcohols and partial aromatic compounds), W5S (nitrogen oxides), and W6S (hydrides) displayed high response values. W2S and W6S responses were higher than those at 1-day storage, which correlates with aroma development throughout fermentation and subsequent cold storage [52]. The radar chart analysis reveals that in yogurts refrigerated for one day, the intensity of two key flavor components, W5S and W2S, showed an increasing trend with higher egg white content (Figure 6B). In contrast, after 14 days of refrigeration, four flavor components—W1S, W2S, W5S, and W6S—exhibited a decreasing trend, which aligns with the patterns observed in the corresponding loading plots. Bai et al. identified aromatic benzene compounds and nitrogen oxides as key volatile flavor components in yogurt, consistent with our findings [53].

As shown in Figure 6C, in yogurts refrigerated for 1 day, the addition of egg white led to decreased richness and sourness values, with slight increases in saltiness and astringency. The observed changes in sourness were consistent with the measured pH values, whereas bitterness, umami and aftertaste remained largely unaffected, suggesting that egg white addition can selectively modulate the sensory attributes of yogurt. After 14 days of refrigeration (Figure 6F), significant changes were observed in the response values of all eight taste sensors, particularly for sourness, which was attributed to increased lactic acid production during cold storage. These taste modifications can be attributed to the high protein content in egg white, which upon hydrolysis generates flavor-active peptides and free amino acids that significantly influence the determining sensory characteristics [54]. These results confirm that egg white addition modifies yogurt’s taste profile, while refrigeration duration predominantly affects sourness intensity. Importantly, egg white incorporation exerts less impact on yogurt’s overall flavor characteristics and does not introduce undesirable off-flavors.

## 3. Conclusions

The experimental results demonstrated that yogurts supplemented with varying levels of egg white exhibited distinct changes in physicochemical properties, texture, and flavor profiles during 1 and 14 days refrigerated storage (4 °C). After 1 day of storage, the 5% egg white yogurt showed significant improvements in physicochemical parameters, textural properties, and rheological performance compared to the control (EW_0_). EW_10_ and EW_0_ exhibited comparable quality attributes, consistent with their similar gel network structures observed by microstructural analysis. However, excessive egg white addition negatively affected all evaluated quality parameters. After 14 days of refrigeration storage, all samples exhibited slight deterioration in both physicochemical and structural characteristics. Interestingly, sensory evaluation revealed remarkable flavor stability throughout storage, with only acidity showing noticeable variation. These findings suggest that moderate egg white addition could effectively enhance short-term yogurt stability while maintaining flavor integrity. Therefore, this study provides theoretical foundations for egg white yogurt research, demonstrating the potential of developing additive-free, high-protein and low-fat egg white yogurt as a novel category of healthy nutritional dairy products. It should be noted that in certain cultural or religious contexts, the consumption of dairy-egg combinations may be restricted. The present work focuses solely on the scientific evaluation of compositional and functional changes, without implying dietary recommendations.

## 4. Materials and Methods

### 4.1. Materials

Fresh hen eggs were procured from a certified poultry farm in Shuangcheng County, Heilongjiang Province, China, and stored under conditions (4 ± 0.5 °C) prior to use. Wonder milk brand pure milk was purchased from Heilongjiang Wonder milk Linhai Liquid Milk Co., Ltd., Harbin, China Granulated white sugar was obtained from Harbin Zhenqizi Food Co., Ltd., Harbin, China The starter culture (YO-MIX 883) was procured from Danisco (Beijing) Trading Co., Ltd., Beijing, China. All other chemical reagents used were of analytical grade and purchased from Harbin Shengda Reagent Company, Harbin, Heilongjiang Province, China.

### 4.2. Preparation of the Egg-White Yogurt

Egg whites were separated and homogenized using a high-speed disperser (T25, LabTech Inc., Beijing, China) to obtain a uniform and stable liquid. The homogenized egg white was incorporated into milk at ratios (5%, 10%, 15%, 20%, and 25% *w*/*w*), followed by the addition of sucrose (3% *w*/*w*). The mixtures were homogenized at 20 MPa and pasteurized at 85 °C for 15 min, cooled to 42 °C before inoculation with starter culture (YO-MIX 883, 0.05% *w*/*w*), samples were fermented at 42 °C for 4 h. The resulting yogurt samples containing 5%, 10%, 15%, 20%, and 25% egg white were designated as EW_5_, EW_10_, EW_15_, EW_20_, and EW_25_, respectively, with egg white-free yogurt serving as the control (EW_0_). All samples were storage at 4 ± 0.5 °C for subsequent analysis.

### 4.3. Color Measurement

The color characteristics were determined using a pre-calibrated portable colorimeter (CM-600d, Konica Minolta Holdings, Inc., Tokyo, Japan), with the L*, a*, and b* values recorded. L* = 0 represents black, and L* = 100 represents white; a higher a* value indicates a closer proximity to red, while a lower a* value indicates a closer proximity to green; a higher b* value indicates a closer proximity to yellow, while a lower b* value indicates a closer proximity to blue. The whiteness index (WI) of the samples was calculated using the following formula [55]:

(1) $$\mathrm{WI}=\;100\;-\;\sqrt{{\left(100-L*\right)}^{2}+{\left(a*\right)}^{2}+{\left(b*\right)}^{2}}$$

### 4.4. pH Measurement

The yogurt samples were equilibrated to 20 °C and the pH was measured using a pH meter (PHS-3C, Shanghai Precision Instrument Co., Ltd., Shanghai, China).

### 4.5. Determination of Titratable Acidity

A 10 g yogurt sample was weighed into a conical flask mixed with 20 mL of freshly boiled and cooled (20 °C) distilled water thoroughly blended. 2 mL of phenolphthalein indicator was added, and the mixture was shaken well. The solution was titrated with 0.1 mol/L NaOH standard solution until a pink endpoint was reached and persisted for at least 5 s. The titratable acidity (TA) expressed as °T was calculated by multiplying the volume (mL) of NaOH standard solution consumed in the titration by 10 [56].

### 4.6. Protein Content Determination

The protein content of yogurt was determined using the biuret colorimetric method using the method adopted from a previous work. Yogurt samples were diluted with 0.067 mol/L phosphate buffered saline (PBS, pH 9.0) and mixed with four volumes of biuret reagent. After incubation at room temperature for 30 min, the absorbance was measured at 540 nm. A standard curve was established using bovine serum albumin standard solutions, yielding the regression equation: y = 0.0482x (R^2^ = 0.9998). The protein concentration (c, mg/mL) was calculated as follows:

c = (*OD_540_*/0.0482) × *N* (2)

where: *OD*_540_ = absorbance at 540 nm; *N* = dilution factor.

### 4.7. Water-Holding Capacity (WHC) Determination

The water-holding capacity (WHC) was centrifuged at 8000× *g* for 15 min at 4 °C, followed by careful removal of the supernatant. The mass of the precipitate was accurately weighed. WHC was calculated using the following equation:

*WHC* (%) = (*M*_2_/*M*_1_) × 100

where *M*_1_ denotes the sample weight, and *M*_2_ denotes the weight after supernatant removal.

### 4.8. Zeta Potential Measurement

The zeta potential of yogurt was measured using a NANO ZS90 Zetasizer (Malvern Panalytical, Malvern, UK). The method of the zeta potential measurement was diluted 100-fold with deionized water before analysis.

### 4.9. Surface Hydrophobicity Determination

ANS (8-anilino-1-naphthalenesulfonic acid) and a fluorescence spectrophotometer (F-7100, Hitachi Corp., Tokyo, Japan) were used to determine the surface hydrophobicity (H^0^) [57]. Samples were diluted with 10 mM phosphate buffer (pH 7.0) to protein concentration of 0.2 mg/mL. 4 mL of the diluted sample was mixed with 20 μL of ANS solution (8.0 mmol/L) excitation wavelength was set at 390 nm emission spectrum was recorded from 400 to 600 nm with both excitation and emission slit widths set to 5 nm [58].

### 4.10. Texture Profile Analysis

The textural characteristics of yogurt were measured using a TA-XT plus texture analyzer (Stable Micro System, Surrey, UK). A 35 mm diameter cylindrical probe was used for texture profile analysis (TPA) [59]. Pre-test and post-test speeds were set at 5.0 mm/s. During testing, the probe penetrated the samples to a depth of 15.0 mm at a constant speed of 5.0 mm/s, with a trigger force of 10.0 g.

### 4.11. Rheological Characterization

The rheological behavior of yogurt samples was assessed using a rotational rheometer (MARS40, HAAKE, Vreden, Germany). Using a 35 mm diameter plate, under conditions of 25 °C, and temperature equilibrium time of 150 s [60]. The shear viscosity was measured under linearly increasing shear rates ranging from 0.01 to 100 s^−1^. Frequency sweep tests were conducted within the linear viscoelastic region at a constant strain amplitude of 0.1% over an angular frequency range of 0.1–10 Hz. The storage modulus (G′) and loss modulus (G″) were recorded.

### 4.12. Fourier Transform Infrared Spectroscopy (FTIR) Analysis

Fourier-transform infrared (FTIR) spectra were acquired using a Nexus 470 spectrometer (Thermo Nicolet Co., Ltd., Waltham, MA, USA). Spectral measurements were conducted at 25 °C with spectral collection ranging from 4000 to 400 cm^−1^ at 4 cm^−1^ resolution, accumulating eight scans per sample. Following initial correction using OMNIC software 9.2, the amide I band (1600–1700 cm^−1^) was analyzed with PeakFit software 4,12 (SPSS Inc., Chicago, IL, USA).

### 4.13. Microstructure Observation

The microstructure of the samples was observed using a field-emission scanning electron microscope (SU8010, Hitachi, Ltd., Tokyo, Japan). All samples were pre-frozen using liquid nitrogen [61]. The frozen samples were transferred into the preparation chamber and fractured using a cold scalpel blade at −177 °C. The fractured sample was then etched at −85 °C for 15 min and coated with 300 A of sputtered gold. Each sample was scanned and imaged at different viewing fields under accelerating voltages of 1 kV and 5 kV.

### 4.14. Electronic Nose Measurements

The flavor profile of yogurt was determined by an electronic nose (e-nose) (SA402B, INSENT Inc., Fukushima, Japan). 10 g post-ripened yogurt was weighed into 20 mL sample via. samples were equilibrated at room temperature for 2 h prior to measurement, according to the methodology described by Mortazavian et al. [62]. All sample groups were analyzed in parallel.

### 4.15. Electronic Tongue Measurements

Electronic tongue measurements were performed according to previously published methods with appropriate modifications [15]. The yogurt samples were centrifuged at 5000× *g* for 15 min, and the supernatant was collected in specialized electronic tongue measurement cups. The supernatant was diluted with distilled water and equilibrated for 1 h prior to analysis.

### 4.16. Statistical Analysis

All experiments were performed in triplicate and results were expressed as mean ± standard deviation (Mean ± SD). Statistical analysis was conducted using SPSS 27.0 (IBM Corp., Armonk, NY, USA), one-way analysis of variance (ANOVA) with a significance threshold set at *p* < 0.05. Duncan’s multiple range test was applied to determine significant differences among groups. Graphical representations were generated using OriginPro 2021 (OriginLab Corp., Northampton, MA, USA).

## Figures and Tables

**Table 1:** Effect of egg white addition on the color parameters of yogurts after 1-day refrigerated storage.

Egg White Addition/%	L*	a*	b*	WI
0	89.43 ± 0.38 ^e^	−0.60 ± 0.01 ^f^	0.95 ± 0.09 ^f^	89.37 ± 0.38 ^d^
5	90.78 ± 0.09 ^d^	−0.45 ± 0.00 ^e^	2.24 ± 0.07 ^e^	90.50 ± 0.07 ^c^
10	94.66 ± 0.15 ^c^	−0.38 ± 0.01 ^d^	2.56 ± 0.06 ^d^	94.06 ± 0.11 ^b^
15	96.08 ± 0.26 ^b^	−0.36 ± 0.00 ^c^	3.53 ± 0.08 ^c^	95.71 ± 0.21 ^a^
20	96.86 ± 0.26 ^a^	−0.31 ± 0.00 ^b^	3.78 ± 0.03 ^b^	95.07 ± 0.17 ^a^
25	97.27 ± 0.10 ^a^	−0.28 ± 0.01 ^a^	4.13 ± 0.03 ^a^	95.05 ± 0.03 ^a^

Data with different superscript letters indicate statistically significant differences (*p* < 0.05).

**Table 2:** Effect of egg white addition on the color parameters of yogurts after 14-day refrigerated storage.

Egg White Addition/%	L*	a*	b*	WI
0	99.37 ± 0.01 ^a^	−0.15 ± 0.00 ^b^	0.09 ± 0.02 ^b^	99.34 ± 0.01 ^a^
5	99.36 ± 0.00 ^a^	−0.14 ± 0.01 ^ab^	0.10 ± 0.01 ^b^	99.34 ± 0.00 ^ab^
10	99.36 ± 0.01 ^a^	−0.14 ± 0.01 ^ab^	0.10 ± 0.01 ^b^	99.34 ± 0.01 ^ab^
15	99.37 ± 0.01 ^a^	−0.14 ± 0.00 ^ab^	0.11 ± 0.02 ^ab^	99.34 ± 0.01 ^ab^
20	99.36 ± 0.01 ^a^	−0.14 ± 0.01 ^ab^	0.11 ± 0.01 ^ab^	99.34 ± 0.00 ^ab^
25	99.36 ± 0.00 ^a^	−0.13 ± 0.01 ^a^	0.13 ± 0.01 ^a^	99.33 ± 0.00 ^a^

Data with different superscript letters indicate statistically significant differences (*p* < 0.05).

## Data Availability

All data and materials are available on request from the corresponding author. The data are not publicly available due to ongoing researches using a part of the data.
